# Trends in guideline implementation: a scoping systematic review

**DOI:** 10.1186/s13012-015-0247-8

**Published:** 2015-04-21

**Authors:** Anna R Gagliardi, Samia Alhabib, and the members of the Guidelines International Network Implementation Working Group

**Affiliations:** Toronto General Research Institute, University Health Network, Toronto, Canada; Department of Family & Community Medicine, King Abdullah University Hospital, Riyadh, Saudi Arabia

**Keywords:** Guidelines, Implementation, Implementation planning, Implementation strategies, Quality improvement, Systematic review

## Abstract

**Background:**

There is currently no reliable way to choose strategies that are appropriate for implementing guidelines facing different barriers. This study examined trends in guideline implementation by topic over a 10-year period to explore whether and how strategies may be suitable for addressing differing barriers.

**Methods:**

A scoping systematic review was performed. MEDLINE and EMBASE were searched from 2004 to 2013 for studies that evaluated the implementation of guidelines on arthritis, diabetes, colorectal cancer and heart failure. Data on study characteristics, reason for implementation (new guideline or quality improvement), implementation strategy used, rationale for selecting that strategy and reported impact were extracted and summarized. Interventions were mapped against a published taxonomy of guideline implementation strategies.

**Results:**

The search resulted in 1,709 articles; 156 were retrieved and 127 were excluded largely because they did not evaluate guideline implementation, leaving 32 eligible for review (4 arthritis, 3 colorectal cancer, 21 diabetes, 4 heart failure). Six of 7 randomized trials and 8 of 25 observational studies had a low risk of bias. Most studies promoted guideline use for quality improvement (78.0%). Few studies rationalized strategy choice (18.8%). Most employed multiple approaches and strategies, most often educational meetings and print material for professionals or patients. Few studies employed organizational, financial or regulatory approaches. Strategies employed that were unique to the published taxonomy included professional (print material, tailoring guidelines, self-audit training or material) and patient strategies (education, counselling, group interaction, print material, reminders). Most studies achieved positive impact (87.5%). This did not appear to be associated with guideline topic, use of theory or barrier assessment, or number or type of implementation approaches and strategies.

**Conclusions:**

While few studies were eligible, limiting insight on how to choose implementation strategies that address guideline-specific barriers, this review identified other important findings. Education for professionals or patients and print material were the most commonly employed strategies for translating guidelines to practice. Mapping of strategies onto the published taxonomy identified gaps in guideline implementation that represent opportunities for future research and expanded the taxonomy.

**Electronic supplementary material:**

The online version of this article (doi:10.1186/s13012-015-0247-8) contains supplementary material, which is available to authorized users.

## Background

Guidelines are considered an essential “foundation” for healthcare policy, planning, delivery, evaluation and quality improvement by clinicians, managers and policy-makers [1]. As noted in the Institute of Medicine report Clinical Practice Guidelines We Can Trust, guidelines translate the complexity of scientific research findings into recommendations that can enhance healthcare quality and outcomes [2]. Despite widespread recognition of their crucial function, guidelines are not always translated to policy or practice [3-5]. Limited use of guidelines contributes to omission of beneficial therapies, preventable harm, suboptimal patient outcomes or experiences or waste of resources [6,7]. This is, in part, a result of multiple factors that often interact to challenge guideline implementation and use and are commonly and collectively referred to as “barriers” to change or more broadly as “determinants of practice” in the guideline implementation literature [8-10]. These include characteristics of the recommended practice and patient, provider, institutional and system-level factors. It is considered important to identify barriers and choose and tailor implementation strategies accordingly so that impact can be optimized. A systematic review of 26 randomized controlled trials found that interventions that had been selected and tailored to address identified barriers were more likely to improve professional practice compared with either no intervention or dissemination of guidelines [11]. However, most studies included in the review provided little information or justification about how interventions were chosen and tailored, so the review concluded that there was insufficient evidence on the most effective approaches for doing so.

Guidance or tools for identifying barriers and choosing interventions have been developed to support guideline implementation planning. For example, guideline implementation taxonomies are available, and numerous systematic reviews have synthesized primary research on the effectiveness of guideline implementation strategies [12,13]. Krause et al. evaluated different methods for identifying barriers and found that brainstorming by the team responsible for guideline implementation and interviews with health professionals identified the largest number of barriers. Interviews with patients identified many different barriers, so the authors concluded that a combination of methods should be used [14]. Another striking finding from the same study was the identification of 601 discrete barriers by the participants from five European countries, underscoring the complex and challenging nature of implementation planning. A number of checklists or questionnaires have been developed to assess organizational readiness to change, implementation capacity or factors that influence the adoption of innovations [15-17]. For example, Michie et al. generated the Theoretical Domains Framework by compiling 12 behaviour change domains reflecting 14 motivational, 11 action and 8 organizational theories which can be used to design implementation interventions [18]. However, these resources do not offer definitive guidance on which implementation strategies best address particular barriers. For example, the BARRIERS scale was found to be reliable when tested, but a systematic review of 63 studies in which it was used showed that information about identified barriers was not used to select or tailor interventions, and in many studies, desired changes were not achieved [19].

Intervention mapping is a five-step process that is meant to support the selection of interventions that address identified barriers [20]. It is a methodological framework to guide implementation planning once a needs assessment has been undertaken that describes a healthcare problem and its cause, including barriers and the capacity of stakeholders to support the desired change. In step one, objectives are established given the identified barriers, stakeholder characteristics and desired outcome. In step two, a list of potential interventions is created through brainstorming, searching the empirical literature to identify the effectiveness of those interventions and deciding how to apply the interventions chosen to match objectives. However, the choice of intervention is subjective and may not necessarily be the most appropriate solution for a given scenario. While a variety of tools and methods exist by which to identify implementation barriers and potentially relevant interventions, there is currently no reliable way to match an intervention to identified implementation barriers. Guideline developers and users have expressed the need for guidance by which to choose, tailor and operationalize implementation strategies, and empirical research has also demonstrated that users are often aware and accepting of guidelines but struggle with their implementation [8-10,21]. Further research is needed to develop objective mechanisms by which to choose implementation strategies that match identified barriers. This would complement and extend the usefulness of existing tools and processes and provide implementation support to guideline developers, implementers and users. Ultimately, this may improve the implementation and use of guidelines, leading to improved healthcare planning, delivery, efficiency and outcomes.

As a preliminary step in establishing a means by which to match interventions with barriers of guideline implementation and use, it would be useful to explore trends in implementation for guidelines on different topics. This may reveal whether particular strategies are feasible and effective for guidelines whose use might be challenged by factors unique to the users or environment within which they are applied. If such trends are identified, they may lead to the development of a matrix or other framework for matching implementation strategies to barriers. If instead gaps in research are identified, that would establish avenues for ongoing primary research. The purpose of this study was to examine published, peer-reviewed research on guideline implementation to describe the strategies that were used and identify trends in use over time or by clinical topic which may suggest implementation strategies that suit different barriers or circumstances.

## Methods

### Approach

We conducted a scoping review of studies that evaluated guideline implementation or use [22]. A scoping review examines whether and how different types of strategies were operationalized and studied. A scoping review also identifies gaps in the literature that can only be addressed through ongoing research. While similar in rigour to a traditional systematic review, a scoping review addresses broader, more complex topics where different study designs may be relevant and selection criteria are developed *post hoc* based on increasing familiarity with the literature. The Preferred Reporting Items for Systematic Reviews and Meta-Analyses (PRISMA) criteria guided reporting of the methods and findings (Additional file 1) [23]. A protocol for this review was not registered.

### Search

MEDLINE and EMBASE were searched from 2004 to 2013 inclusive for studies that evaluated guideline implementation planning, processes or outcomes. A 10-year time span was used as, over this period, recognition of the need for tailoring the design of implementation strategies according to barriers or determinants emerged, so studies published later in the time frame may have reflected this. These are key indexed databases featuring the largest volume of published medical research. Searches were executed in January 2014 and updated in June 2014 to ensure that we captured all studies published in 2013. The search strategy was purposefully broad to be as inclusive as possible (Additional file 2). Search terms were informed by research that generated search strategies for implementation topics which optimized sensitivity and specificity and included Medical Subject Headings and keywords [24,25]. Guideline topics included arthritis, colorectal cancer, diabetes and heart failure. These conditions affect both men and women and are major causes of disability and death worldwide. Furthermore, they reflect both primary (arthritis, diabetes) and acute (colorectal cancer, heart failure) settings of care to account for differing institutional and system-level factors, involve different types of providers and patients and recommend different processes of care, thereby addressing remaining factors known to challenge guideline implementation and use [7-9]. Focusing on a few topics also enhanced feasibility of conducting this scoping review. Searches were limited to English language to avoid the cost of translation.

### Screening

Titles and abstracts of search results were reviewed independently by both authors and a research assistant. All items selected by at least one reviewer were retrieved for further assessment since judgment about eligibility must often be reserved until the full text can be reviewed. If more than one publication described a single study and each presented the same data, the most recent was included. Preliminary selection criteria included observational studies, randomized trials, case studies or programme evaluations that described the methods used to implement new guidelines or promote compliance with guidelines on relevant topics. As search results were reviewed, selection criteria were expanded to specify studies that were not eligible. These included studies that evaluated clinical interventions for patient care or behavioural interventions for an issue other than guideline implementation; condition-specific prevalence, risk factors or complications; practice patterns based on audits or population-based data; barriers or facilitators influencing guideline adherence; guideline development strategies; or strategies targeted to trainees only or where the strategy was not sufficiently described. Guidelines, anecdotal reports, protocols, abstracts, letters, commentaries or editorials were not eligible.

### Data collection and analysis

A data extraction form was developed to capture information from each article on year of publication, country of origin, study design, guideline topic, reason for implementation (new guideline or quality improvement based on an existing guideline), implementation strategy or strategies used, theory or identification of barriers used to inform the selection or tailoring of implementation strategy and impact according to measures reported in individual studies. The implementation strategy was described according to content, mode, duration, frequency, audience and personnel. Implementation strategies were labelled according to the Mazza et al. taxonomy which categorizes 49 distinct strategies as higher level professional, organizational, financial and regulatory approaches [12]. For example, professional approaches included the strategy of “advertise guideline material”, and organizational approaches included the strategy of “reallocated or new role” targeted to health professionals and the strategy of “consumer participation in governance” targeted to patients. This mapping strategy also identified strategies employed in eligible studies but not listed in the Mazza et al. taxonomy. The form was pilot tested independently by ARG and a research assistant in two iterations for three randomly selected articles to achieve high congruence of data extraction. Data were independently extracted by two research assistants and then checked for accuracy by ARG. Study quality was assessed using criteria relevant to study design, but was not used to exclude studies. This included the Cochrane Risk of Bias tool for randomized controlled trials and a modified version of the Downs and Black instrument for observational studies [26,27]. Search results were depicted with a PRISMA flow diagram [23]. Summary statistics were used to describe the number of articles by topic, country, year of publication, reason for implementation and those in which the intervention was informed by theory or otherwise rationalized. The number of implementation strategies was summarized by implementation approach and guideline topic. The number of single or multiple approaches and strategies was also summarized. Tabulated data were observed for trends or possible links between guideline topic (which reflected barriers relevant to different types of care processes, providers, patients and settings), reason for implementation and type and number of implementation approaches or strategies. Studies included a wide range of designs and measures, and pooling of data was not possible. Impact was described as the number of studies by topic and type of implementation strategy that achieved intended outcomes.

## Results

### Characteristics of eligible studies

**Figure 1:**
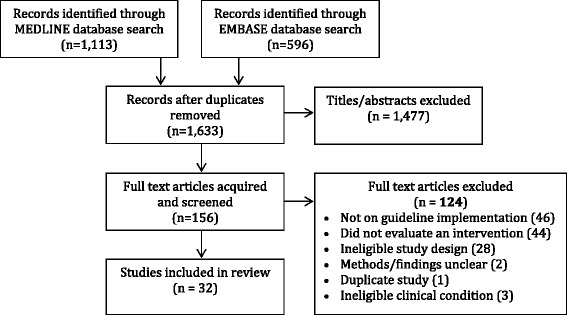
PRISMA flow diagram of search results.

The initial search resulted in 1,709 articles. Following removal of duplicates 1,477 titles were not eligible, and 156 items were retrieved as potentially relevant. Of these, 127 articles were excluded because they did not examine guideline implementation (46) or evaluate an intervention (44), study design was ineligible (28), methods or findings were unclear (2) and publication was a duplicate (1) or not focused on the conditions of interest (3) leaving 32 studies eligible for review (Figure 1). Study details appear in Additional file 3 [28-59]. Most were based in primary or community care settings (22, 68.8%). Seven eligible studies were randomized controlled trials and 25 were observational in design. Not counting 2007 in which there were no eligible studies published, three studies per year were published on average (range two in 2011 to six in 2013). Most studies were conducted in the United States (16) while others were based in Canada (4), Germany (2) and the Netherlands (2), and individual studies were undertaken in the United Kingdom, Africa, Austria, Australia, Italy, Spain and Finland. Most studies focused on diabetes (21) with few studies on arthritis (4), colorectal cancer (3) and heart failure (4). Quality assessment results appear in Additional file 4. Of seven randomized controlled trials, six had a low risk of bias and one had a high risk of bias. Of 25 observational studies, 6, 16 and 3 had a low, moderate and high risk of bias, respectively.

### Implementation approaches

The majority of studies were undertaken to promote compliance with existing guidelines for quality improvement (25); fewer studies implemented a newly developed or updated guideline (7). Of 32 eligible studies, 6 (5 quality improvement, 1 new guideline implementation) explicitly described a rationale for the implementation strategy used (2 arthritis, 2 colorectal cancer, 2 diabetes) by referring to models or theories including the Knowledge to Action cycle [28], Chronic Care Model [43,51], Precaution Adoption Process Model [33], Social Cognitive Theory [29,34] and the Theory of Reasoned Action [34]. Two diabetes studies referred to other rationale for the implementation strategy used. One of these studies mentioned the identification of barriers, but not how this was done [48], and the other mentioned that the guideline was tailored, but not why or how [49]. No other studies explicitly noted whether or how barriers were identified or whether barriers influenced the selection or tailoring of implementation strategies.

Implementation approaches and strategies used in eligible studies and described according to the Mazza et al. taxonomy [12] appear in Table 1. Strategies not already listed as financial and organizational approaches that were targeted to professionals (print material, tailoring guidelines, self-audit training or material) and to patients (education, counselling, group interaction, print material, reminders) appear in Table 1 as italicized text. Few studies used financial or organizational approaches, and no studies used regulatory approaches. The majority of studies employed professional approaches, most commonly educating groups about guideline intent and benefits, reminding individuals or groups about guideline intent and benefits and providing print material such as summaries, algorithms or referral forms. Approaches and strategies not listed in the Mazza et al. taxonomy but identified in eligible studies were added in italicized text to the bottom of the table. Numerous studies also offered patient or consumer approaches, most commonly educating groups about guidelines, counselling on lifestyle issues or self-management and print material such as guideline summaries.

**Table 1:** **Implementation approaches and strategies used in eligible studies**

**Strategy** ^**a**^	**Arthritis (4)**	**Colorectal Cancer (3)**	**Diabetes (21)**	**Heart Disease (4)**	**Total**
Professional					
Identify barriers	--	--	2	--	2
Distribute guideline material	--	--	2	--	2
Advertise guideline material	--	--	--	--	0
Present guideline materials at meetings	--	--	--	--	0
Educate individuals about guideline intent/benefits	--	1	2	--	3
Educate groups about guideline intent/benefits	2	--	13	3	18
Recruit an opinion leader who recommends implementation	--	--	--	--	0
Achieve consensus that guideline should be implemented	--	--	--	--	0
Provide reminders to individuals/groups about intent/benefits	2	1	7	1	11
Provide alerts when practice deviates	--	--	--	--	0
Provide feedback on compliance	--	1	1	1	3
Provide feedback about patients (outcome data, self-report)	--	--	3	--	3
Provide feedback from patients	--	--	--	--	0
Provide feedback from healthcare professionals	--	--	--	--	0
*Print material (summary, algorithm, referral forms, etc.)*	2	3	6	2	13
*Tailor guideline*	--	--	1	--	1
*Enable self-audit (training, material)*	--	--	1	--	1
Subtotal	6	6	38	7	57
Financial					
Health professional					
Incentive (individual financial reward or benefit for compliance)	--	--	--	--	0
Incentive (group or institutional financial reward or benefit)	--	--	--	--	0
Grant or allowance to individual (not tied to compliance)	--	--	4	--	4
Grant or allowance to group/institution (not tied to compliance)	--	--	--	1	1
Penalty (individual, for non-compliance)	--	--	--	--	0
Penalty (group/institution, for non-compliance)	--	--	--	--	0
Change in reimbursement (add/remove/substitute)	--	--	--	--	0
Patient					
Incentive (individual financial reward/benefit for compliance)	--	--	--	--	0
Grant or allowance (not tied to compliance)	--	--	--	--	0
Penalty (for non-compliance)	--	--	--	--	0
Incentive (individual non-financial reward/benefit for compliance)	**--**	**--**	1		1
Subtotal	0	0	5	1	6
Organizational					
Health professional					
Additional human resources (number/type)	--	--	2	--	2
Reallocated or new role	--	--	1	--	1
Create an implementation/multidisciplinary team	--	--	1	--	1
Communication between distant health professionals	--	--	--	--	0
Improve health professional satisfaction (non-financial)	--	--	--	--	0
Patient					
Consumer participation in governance	--	--	--	--	0
Consumer feedback, suggestions, complaints	--	--	--	--	0
Structural changes					
Organizational structure (including reorganization)	--	--	--	--	0
Setting/site of service delivery	--	--	--	--	0
Physical structure, facilities or equipment	--	--	--	--	0
Information/communication technology	--	--	3	1	4
Quality improvement, performance measurement system	--	--	--	--	0
Method of service delivery	--	--	--	--	0
Integration of services	--	--	1	--	1
Risk management provisions (including insurance coverage)	--	--	--	--	0
Subtotal	0	0	8	1	9
Regulatory					
Legislation or regulation (which enforces or mandates)	--	--	--	--	0
Ownership or affiliation	--	--	--	--	0
Licensing, credentialing or accreditation	--	--	--	--	0
Subtotal	0	0	0	0	0
*Patient/consumer*					
*Education (single or group)*	1	--	7	1	9
*Counselling*	--	1	4	1	6
*Group interaction (via social media)*	1	--	--	--	1
*Print material (summary, etc.)*	--	1	5	1	7
*Reminder*	--	1	1	--	2
Sub-total	2	3	17	3	25
Total	8	9	68	12	97

^a^Definitions for/examples of strategies included in Additional file of Mazza et al. taxonomy publication [11]; italicized items emerged from this study and were not included in the Mazza et al. taxonomy.

Five studies (one arthritis [30], one colorectal cancer [32], three diabetes [37,46,54]) employed a single-implementation approach based on a single strategy, for example, professional education. Four diabetes studies employed multiple implementation approaches, each comprised of a single strategy [35,39,42,53], for example, a professional approach involving reminders combined with an organizational approach involving a new professional role. Fourteen studies (three arthritis [28,29,31], two colorectal cancer [33,34], five diabetes [36,38,41,44,48], four heart failure [56-59]) employed a single-implementation approach comprised of multiple strategies, for example, a professional approach involving education via workshop, print material and a reminder. Nine diabetes studies employed interventions comprised of multiple approaches where at least one of the approaches involved multiple strategies [40,43,45,47,49-52,55]. With respect to topic, diabetes studies appeared to employ more strategies across all approaches (70.1%) compared with arthritis (8.2%), colorectal (9.3%) and heart disease studies (12.4%) though this may reflect the higher number of eligible studies based on diabetes guidelines.

Overall, there were no observable trends in implementation approaches or strategies by setting (primary/community care or otherwise) or by guideline topic since the most frequently employed strategies were education, reminders and print material for professionals, and education, print material and counselling for patients across all topics. The types of implementation approaches and strategies did not appear to change over time. Two arthritis [28,29] and two colorectal [33,34] studies that employed theory both used single approaches involving multiple strategies though the approaches and strategies varied. Two diabetes studies that employed theory, the chronic care model in both cases, used multiple approaches and strategies which differed in the two studies [43,51]. The diabetes study that identified barriers used a single approach with multiple strategies [48], and the diabetes study that tailored a guideline used multiple approaches and strategies [49].

### Impact on knowledge, behaviour and outcomes

All four arthritis studies achieved positive impact on patient and professional knowledge and behaviour. Two of three colorectal cancer studies achieved mixed results. In one study that involved distribution of a referral form to general practitioners, use of the form and appropriate use of the form were variable; however, compared with patients referred as usual, use of the referral form was associated with higher rates of cancer detection [32]. In another study involving telephone counselling, a reminder and print material, 39% of eligible patients in the observational cohort were compliant with screening recommendations [33]. In the third colorectal cancer study which used a randomized controlled trial (RCT) design to test a professional approach involving educational outreach, feedback, print material and a reminder, follow-up of abnormal faecal occult blood test results improved compared with the control group [34]. Of 21 diabetes studies, 19 studies achieved positive impact in measures assessed. Diabetes and hypertension control did not change in 9 intervention sites compared with 9 control sites following professional educational outreach, print material and a reminder [38] or among 50 physicians who received education, print material and reminders plus print material for patients compared with 49 control physicians [40]. Three of four heart failure studies achieved positive impact. In one study involving professional education, print material and feedback prescribing behaviour and patient outcomes such as readmission and morbidity improved [57]. In an RCT testing use of a structural approach involving an information system, reimbursement and training of physicians, recommendations for rehabilitation improved among intervention hospitals compared with control hospitals [58]. Another heart failure study involving patient education, counselling and print material improved their knowledge, behaviour and outcomes [59]. A fourth study did not improve use of stress echocardiography from baseline after professional education, print material and a reminder [56]. While most studies (28/32, 87.5%) achieved positive impact, they employed a variety of types and numbers of approaches and strategies; studies that employed a single approach and strategy and achieved positive impact were few, and all four employed a different strategy [30,37,46,54], so it is difficult to discern the impact of single or combined approaches or strategies. With respect to the group of six studies that used theory-informed strategies, one did not achieve the desired impact. This was a colorectal study that failed to improve motivation for screening [33]. The study that assessed barriers improved diabetes care delivery for 50% of indicators measured [48]. Study design and risk of bias did not appear to be associated with intervention impact.

## Discussion

Numerous strategies have been used to implement guidelines, but their impact is inconsistent. There is currently no reliable way to choose strategies that are most appropriate for implementing guidelines to address determinants of practice in different contexts. To generate knowledge that would provide guidance for choosing and tailoring implementation strategies, this study examined trends in guideline implementation over a 10-year period to identify whether and how strategies and their impact differed by guideline topic as a proxy for context-specific determinants. Overall, few studies were identified, and most focused on the implementation of diabetes guidelines, so the identification of trends in guideline implementation by comparing the use and impact of strategies by guideline topic was not possible. Most studies employed either professional or patient education though these were often combined with other strategies. Choice of strategy did not appear to be associated with guideline topic or change over time. The majority of studies achieved positive impact on patient and professional knowledge and behaviour and on patient outcomes. This did not appear to be associated with guideline topic, use of theories or barrier assessment, or number or type of implementation approaches and strategies.

Few studies reported selecting or tailoring implementation strategies based on theory, assessing barriers or any other rationale. This finding also emerged in other systematic reviews on implementation [60,61] despite the existence of reporting standards that require a justification for strategy selection and clear description of intervention design [62-64]. Therefore, it could not be ascertained if this was associated with choice or tailoring of strategy or impact. Moreover, this did not change over time despite increasing awareness of the need to assess barriers or determinants and tailor strategies according to these factors [10] and a plethora of systematic reviews, taxonomies, theories, models and instruments for doing so [11-18]. Such resources may not be easy to use or broadly relevant [19] and do not objectively or reliably suggest appropriate implementation strategies, so further research is needed to generate guidance for identifying barriers and choosing implementation strategies suitable for addressing those barriers. The responsibility for implementation planning, of which barrier assessment is a part, also remains unclear. Most of the studies aimed to improve compliance with guidelines in the context of quality improvement rather than implementing new guidelines, and in most studies, personnel were either not specified or were health professionals. None of the studies explicitly mentioned involving knowledge brokers or facilitators, individuals with expertise in implementation, in the planning or delivery of interventions [65]. Our group recently generated a framework for implementation planning which also recommends that individuals with expertise in implementation be involved in guideline development from the outset [66].

This study and other research showed that most often guidelines are implemented using educational approaches such as workshops [13,21]. This is not surprising because, in comparison with more complex interventions such as organizational, financial or regulatory strategies which were rarely used by studies included in this review and require large-scale change and/or considerable funding, educational approaches may be less resource intensive and more easily employed by guideline developers or implementers with limited funding [21]. Educational meetings are known to have a small impact on practice [67]. Most studies included in this review achieved desired impact even though they did not report using theories or barrier assessment to inform strategies and employed educational materials and meetings directed at health professionals or patients. This may be due to the fact that educational approaches were often combined with other approaches and strategies. The use of single versus multiple implementation approaches and strategies remains controversial. For example, a recent systematic review found that multiple strategies were more likely to achieve beneficial outcomes, in particular when they combined strategies meant to reach many individuals in a wide variety of settings (post, email, electronic, mass media), motivate interest in the evidence (champions, opinion leaders, social networks) and enhance ability to apply the evidence (additional information or tools that show how to incorporate the evidence into practice) [68]. Yet another rigorous meta-review of 25 systematic reviews that compared direct and indirect effect size and dose–response of single and multifaceted strategies showed no benefit of multifaceted over single strategies [69].

Implementation approaches and strategies were categorized according to the Mazza et al. taxonomy of guideline implementation strategies [12]. It was generated by mapping strategies employed by those whose research was presented in posters or presentations at conferences hosted by the Guidelines International Network onto the Effective Practice and Organisation of Care (EPOC) group’s data collection checklist [70]. The Mazza et al. taxonomy was easy to apply, and most strategies used in eligible studies could be readily categorized according to its components. This research added to the Mazza et al. taxonomy by identifying additional professional (print material, tailored guideline, self-audit) and patient (education, counselling, social networks, print material, reminders) strategies employed in eligible studies. The extended taxonomy that appears in Table 1 can be used by guideline developers, implementers, users or researchers to plan and undertake implementation. Another useful resource that identified and defined 68 discrete strategies for implementing not only guidelines, but a range of evidence-based health innovations, was generated by Powell et al. who compiled a framework from the components of 41 reviews published between 1995 and 2011 [71]. This was further refined and expanded to 73 discrete implementation strategies using a three-round expert Delphi process [72].

Print material aimed at both professionals and patients emerged as a commonly used strategy distinct from the distribution of guideline material which was already included in the Mazza et al. taxonomy. Research on print material offers conflicting evidence of their impact. For example, a systematic review found that print material such as medical journals or guidelines have a small impact on provider behaviour [73]. Yet, other research shows that providers and patients are most likely to benefit from guidelines that offer summaries or other tools that support guideline implementation [68], and experts have called upon those who generate guidelines to develop implementation tools [6,7]. International developers that we interviewed said that they required guidance for developing implementation tools [21]. Our analysis of instructional manuals for developing and implementing guidelines found an absence of such guidance [74]. Therefore, we consulted with members of the international guideline community to generate criteria, methods and considerations for developing guideline implementation tools [75,76].

This study features both strengths and limitations. This study is unique among other systematic reviews that examined the effectiveness of particular implementation strategies [13]. In comparison, this study attempted to identify the types of strategies that were used to implement guidelines on particular topics that may be challenged by unique barriers. Most studies provided sufficient detail about interventions such that the implementation approaches and strategies could be readily ascertained. Screening and data extraction were undertaken independently by multiple individuals to optimize reliability. However, several issues may limit the interpretation and use of these findings. Few studies were eligible, limiting the comparison of implementation approaches and strategies across clinical topics and limiting the pooling of data to assess determinants of strategies used or the impact of those interventions. Other systematic reviews of guideline implementation included a larger number of studies but spanned many more years and healthcare diseases or issues [13,60]. Of 32 eligible studies, 14 were assessed to have a low risk of bias. Since more than half of the studies had a moderate or high risk of bias, findings must be interpreted with some caution. Although we searched the two most relevant medical databases, the literature search may not have identified all relevant studies. We did not search the grey literature, assuming that most empirical research on guideline implementation would be found in indexed databases. Publication bias, or the tendency for journals to publish positive results, may have influenced the number and type of studies that were retrieved. No trends in implementation over time or by guideline characteristics were identified. Since the examination of guideline characteristics was based on guideline topic (hence, indirectly on guideline users), it was therefore limited to details that were reported in eligible studies. Other guideline characteristics may be relevant, but their influence on use and reported impact of implementation strategies would need to be assessed through primary data collection. The focus on four guideline topics may also have limited the ability to identify trends in the use and impact of guideline implementation strategies, so future research could repeat this study for additional guideline topics. However, given the limited yield of this scoping review, it is unclear whether similar reviews on additional clinical topics are warranted. Instead, rigorous primary research should be undertaken to optimize the design of interventions that would ultimately offer insight on the ideal implementation approaches and strategies. Such research may pursue two broad themes. This review identified numerous approaches and strategies that have not been used to implement guidelines on the topics examine in this study but warrant further investigation. This review also identified that, despite the availability of evidence, taxonomies, theories, models and instruments by which to plan implementation, guidelines are most often implemented using educational strategies and print material. Most of the studies reviewed here achieved positive impact, which perhaps conflicts with the results of systematic reviews demonstrating that educational meetings and print material have a small impact on professional behaviour [67,73]. Therefore, ongoing research might focus on ways to optimize the design of educational strategies and print material.

## Conclusions

While few studies were eligible, limiting the identification of trends in guideline implementation and insight on how to choose implementation strategies that address guideline-specific barriers, this review identified other important findings. Education for professionals or patients and print material were the most commonly employed strategy for translating guidelines to practice. Mapping of strategies identified in eligible studies onto the Mazza et al. taxonomy [12] identified gaps in guideline implementation that represent opportunities for future research and expanded the Mazza et al. taxonomy which is a useful tool for classifying implementation strategies. Few interventions were planned based on theory, barrier assessment or other rationale. Most studies employed multiple approaches and strategies and achieved the desired impact. This did not appear to be associated with guideline topic or number or type of implementation approaches and strategies.

## Additional files

Additional file 1: PRISMA checklist. Completed PRISMA checklist indicating page number in manuscript of relevant content. Additional file 2: Literature search strategy. MEDLINE search strategy. Additional file 3: Data extracted from eligible studies by condition of interest. Research design, implementation strategy design and impact of eligible studies by disease site [28-59]. Additional file 4: Methodological assessment of eligible studies. Scoring of randomized control trials and observational studies using the Cochrane Risk of Bias tool and Down’s and Black Checklist, respectively [38-40,42,58,52,34,28,35-37,56,32,41,57,29,43-45,30,48,46,47,49,50,33,53,31,51,54,59,55 **]**.
