# Supplementary material for: Trends in guideline implementation: a scoping systematic review
Source: Implement Sci. 2015 Apr 21;10:54. doi: 10.1186/s13012-015-0247-8 (PMC4409784; doi:10.1186/s13012-015-0247-8)
Supplement: Additional file 2: — Literature search strategy. MEDLINE search strategy. [file 13012_2015_247_MOESM2_ESM.doc]

**Additional File** **2. Literature Search Strategy**

Database: Ovid MEDLINE(R) Search Strategy:

--------------------------------------------------------------------------------

1 Practice Guidelines as Topic/ (74264)

2 guideline adherence/ (19553)

3 use:.mp. (2873616)

4 implement:.mp. (176766)

5 (utilisation or utilization).mp. (85034)

6 adopt:.mp. (98990)

7 disseminat:.mp. (57385)

8 translat:.mp. (146024)

9 intervention.mp. (249891)

10 "diffusion of innovation"/ (11701)

11 2 or 3 or 4 or 5 or 6 or 7 or 8 or 9 or 10 (3345153)

12 1 and 11 (32972)

13 Colorectal Neoplasms/ (46383)

14 arthritis/ or osteoarthritis/ (22445)

15 diabetes mellitus/ or diabetes mellitus, type 2/ (108557)

16 heart diseases/ or myocardial ischemia/ (48602)

17 12 and 13 (321)

18 limit 17 to (english language and yr="2004 - 2013") (243)

19 12 and 14 (85)

20 limit 19 to (english language and yr="2004 - 2013") (55)

21 12 and 15 (931)

22 limit 21 to (english language and yr="2004 - 2013") (645)

23 12 and 16 (273)

24 limit 23 to (english language and yr="2004 - 2013") (170)

.mp. refers to keyword in the Ovid system; all other terms are Medical Subject Headings
