# Supplementary material for: Trends in guideline implementation: a scoping systematic review
Source: Implement Sci. 2015 Apr 21;10:54. doi: 10.1186/s13012-015-0247-8 (PMC4409784; doi:10.1186/s13012-015-0247-8)
Supplement: Additional file 3: — Data extracted from eligible studies by condition of interest. Research design, implementation strategy design and impact of eligible studies by disease site [28-59]. [file 13012_2015_247_MOESM3_ESM.doc]

**Additional File 3. Data extracted from eligible studies by condition of interest**

Arthritis Studies

| Study  Country  Risk of bias  Overall impact | Research Design  Participants  Objective | Implementation Approach | Implementation Design | Impact |
| --- | --- | --- | --- | --- |
| Brosseau 2013 [28]  Canada  Moderate  Positive | Observational  99 patients with arthritis across Canada  Improve knowledge, skill and self-efficacy | Context:  Quality improvement  Intervention:  Patient (education via Internet, social interaction)  Theory:  Knowledge to Action Cycle | Content: Facebook page offered access to two YouTube videos, two PowerPoint presentations on self-management, wall and comment tools enabled patient interaction  Mode: social media, video, PowerPoint  Duration: 3 months  Audience: mean age 56.4 years; 81% female  Frequency: as desired  Personnel: Healthcare professionals narrated the video presentations | - Knowledge improved at two weeks for in-depth (mean difference 0.8), introductory (mean difference 1.0) and combined topics (mean difference 1.8) (p≤0.01) - At three months intention to practice at least one self-management strategy was carried out (statistically significant only for aquatic jogging/therapy and yoga) - Mean self-efficacy scores improved at two weeks, but not statistically significant difference at three months - Mean confidence score at two weeks and three months were not statistically significant |
| Lineker  2011 [29]  Canada  Moderate  Positive | Observational  553 staff, nurses, physicians, rehabilitation professionals from 189 primary care practices  Improve knowledge, skill, self-efficacy, satisfaction and management of arthritis | Context:  New guideline  Intervention:  Professional (education via workshop, print material, reminder)  Theory:  Social cognitive theory | Content: Workshop offering information on arthritis management, hands-on training for musculoskeletal examination, and small group discussion; print material, posters, referral templates, community resource lists; follow-up on personal goals set at workshop  Frequency: single event  Mode: in-person (follow-up not specified)  Duration: 27-1 day workshops across Canada, follow-up at six months  Audience: Interprofessional  Personnel: Arthritis specialists (rheumatologists, dieticians, physical and occupational therapists, social workers, pharmacists) | - Scoring of responses to survey case scenarios on management of early and late rheumatoid arthritis and knee osteoarthritis improved from baseline for nurse practitioners (p<0.05) and rehabilitation therapists (p<0.05) but not for physicians, nurses or other staff - Proportion of respondents choosing best practices increased for education, joint protection, psychosocial support, referral to rheumatology, and nonsteroidal anti-inflammatory drugs and disease-modifying antirheumatic drugs use as indicated - Recommendations for education, healthy eating and weight management improved (all p<0.05) - Satisfaction and confidence improved (p<0.05) and perceived barriers to accessing specialists decreased (p<0.01) |
| Laine  2009 [30]  USA  Low  Positive | Observational  23,504 patients with arthritis from 1,008 sites worldwide  Use of co-therapy in NSAID users with increased gastrointestinal risk | Context:  Quality improvement  Intervention:  Professional (Reminders)  Theory:  Not specified | Content: reminder of co-therapy treatment options  Mode: checklist form of risk factors and co-therapy reminder for patients with risk factors  Duration: single reminder  Frequency: reminder mid-way through intervention period  Audience: Physicians  Personnel: Not specified | Co-therapy in high risk patients increased from 43% to 61% (95% CI 16%,19%) |
| Rahme  2005 [31]  Canada  Low  Positive | Observational  249 general practitioners from 8 towns in Quebec assigned to four groups: workshop only (20 physicians), decision tree only (54 physicians), combined (84 physicians), no intervention (82 physicians)  Improve arthritis treatment and reduce costs | Context:  Quality improvement  Intervention:  Professional (education via workshop, print material)  Theory:  Not specified | Workshop  Content: Management of patients with arthritis  Duration: 90 minutes  Mode: in-person  Frequency: single event  Audience: general practitioners  Personnel: General practitioner and rheumatologist  Decision Tree/Algorithm  Content: Pharmacological and non-pharmacological treatment options based on  patient response to treatment and risk factors  Mode: Laminated sheet  Duration: as needed  Frequency: single distribution  Audience: general practitioners  Personnel: Distribution by sales representatives with explanatory letter from Continuing Medical Education Department | Compared with no intervention group, prescribing improved from baseline:   - 94% with combined strategy (OR=1.5, 95% CI=0.9, 2.3) - 74% with workshop alone (OR=1.3, 95% CI=0.6, 2.4) - 55% decision tree alone (OR=1.1, 95% CI=0.3, 1.6) |

**Colorectal Cancer Studies**

| Study  Country  Risk of bias  Overall impact | Research Design  Participants  Objective | Implementation Approach | Implementation Design | Impact |
| --- | --- | --- | --- | --- |
| Jani  2012 [32]  UK  Moderate  Mixed | Observational  586 referrals from general practitioners to one of six Acute Hospitals Trusts in the Thames Valley Cancer Network  Increase referrals and cancer detection rates | Context:  Quality improvement  Intervention:  Professional (print material)  Theory:  Not specified | Content: proforma showing indications for referral  Mode: print material  Duration: use as needed over 9 months  Frequency: single distribution  Audience: general practitioners  Personnel: Not specified | - Proforma use varied across six Trusts from 18% to 96% - Compliance of referrals with indications ranged from 30% to 50% - Compliance of referrals with indications among those referred by proforma ranged from 50% to 90% - Proforma use was associated with higher cancer detection rates (p=0.03) |
| White  2006 [33]  USA  Low  Mixed | Observational  582 patients from 38 primary care physicians in Massachusetts  Educate patients to motivate screening | Context:  Quality Improvement  Intervention:  Patient (counselling via phone call, reminder, print material)  Theory:  Precaution Adoption Process Model | Content: letter notifying patient of impending phone call, computer-assisted telephone counselling system to promote colorectal cancer screening, follow-up letter with summary of discussion and educational brochure  Duration: mean 29 minutes  Mode: telephone, print material  Frequency: single phone call (maximum 10 attempts, 3 voice-mail messages)  Audience: patients  Personnel: trained telephone counsellors | - 1,025 (87%) of potentially eligible patients were reached and 725 (71%) of those were eligible for counselling - 97 (17%) were not planning any screening 256 (44%) were planning at least one test but their plan was not compliant with recommendations, and 229 (39%) had a screening plan that was compliant with recommendations - Many patients were not prepared to fully commit to future testing/screening physician |
| Myers  2004 [34]  USA  Low  Positive | RCT  2,992 patients from 470 primary care physicians in 318 practices in Southern Pennsylvania and New Jersey  INT (198 practices): reminder, feedback, educational outreach  CON (120 practices): usual care  Increase physician diagnostic evaluation of abnormal fecal occult blood test screening results | Context:  Quality improvement  Intervention:  Professional (educational outreach, feedback, print material, reminder)  Theory:  Social Cognitive Theory and Theory of Reasoned Action | Content: educational outreach by nurse specialist with presentation on diagnostic evaluation and discussion of barriers, feedback on diagnostic evaluation rates, print material (first visit); presentation reviewed, feedback on diagnostic evaluation rates and aggregated data (second visit), tailored letter summarizing physician-specific barriers; phone call to discuss barriers; feedback report  Mode: in-person (visit), mailed letter and reports, telephone  Frequency: second visit 6 months after first, feedback report at 6 and 12 months  Audience: Primary care physicians  Personnel: visits by nurse specialist, phone calls by study co-investigator | INT significantly increased follow-up of abnormal fecal occult blood test compared with CON (OR=1.63, 95% CI 1.06 to 2.50, p=0.03) |

Diabetes Implementation Studies

| Study  Country  Risk of bias  Overall impact | Research Design  Participants  Objective | Implementation Approach | Implementation Design | Impact |
| --- | --- | --- | --- | --- |
| Butala  2013 [35]  USA  Moderate  Positive | Observational  469 patients at the Free Clinic in New Haven, Connecticut (275 pre/194 post)  Improved use of preventive screening | Context:  Quality improvement  Intervention:  Professional (reminder), Organizational (new role)  Theory:  Not specified | Content: Before appointment chart was reviewed to flag indicated preventive health screening, vaccination or other follow-up items not addressed in previous visits  Mode: Reminder in patient chart  Duration: Ongoing since 2010  Frequency: Not specified  Audience: Most patients are immigrants  Personnel: new role for student volunteer of Medical Records Specialist | Adherence to guidelines increased after intervention for three of four services examined: HIV testing (p=0.0035), receipt of fasting lipid panel (p=0.033), and receipt of fasting blood glucose (p=0.0594); Pap testing remained the same |
| Hager  2013 [36]  USA  Moderate  Positive | Observational  48 nursing home residents and all staff in a single facility  Improve diabetes management | Context:  Quality improvement  Intervention:  Professional (education via workshops and self-directed learning, feedback)  Theory:  Not specified | Content: Workshops and self-taught modules on diabetes management; administrative nursing personnel also offered round-table discussions with diabetes educator and certification in diabetes care; staff were reimbursed for time spent on all activities; scorecard placed in patient charts to track diabetes management quality indicators  Mode: in-person, print material  Duration: 3 years  Frequency: 7 hours of classes  Audience: All nursing home staff  Personnel: not specified | - 88% of patients had HbA1C less than 8% within 6 months of admission - 72.9% were appropriately taking coagulants - 73% had recorded lipid levels and 60% of those were prescribed antilipemics of which 36% had low density lipoprotein levels below 100 mg/dL |
| Kuhne-Eversmann  2013 [37]  Germany  High  Positive | Observational  103 general practitioners from Germany  Improve diabetes care knowledge and delivery | Context:  Quality improvement  Intervention:  Professional (education via workshop)  Theory:  Not specified | Content: Workshop on diabetes management including small group case-based exercises  Mode: in person  Duration: 5 hours  Frequency: single session  Audience: Physicians (mean age 48.4, 56% female)  Personnel: not specified | Knowledge-based questionnaire scores improved from 43.9% to 62.6% (p<0.001)  Compared with a control group, referrals to diabetes specialists increased by 30.7% (p<0.001) |
| Steyn  2013 [38]  Africa  Low  Mixed | RCT  Clinicians at 18 community health centres in Cape Town  INT (491 patients at 9 sites): outreach visit plus a structured record  CON (475 patients at 9 sites): outreach visit, no structured record provided  Improve diabetes and hypertension care knowledge and delivery | Context:  New guideline  Intervention:  Professional (educational outreach, print material, reminder)  Theory:  Not specified | Content: educational outreach to review guideline recommendations and train physicians on use of chart resources including checklist reminder, algorithm, and template for recording results of clinical/diagnostic tests, follow-up visits to discuss challenges  Mode: in person, print material  Duration: 9 months  Frequency: 3 outreach visits (2 weeks and 2 months after initial visit)  Audience: general practitioners, nurses  Personnel: Outreach visit by recognized local diabetes and hypertension expert | Fewer than 60% of patient charts contained chart resources, and diabetes and hypertension control did not change |
| Flamm  2012 [39]  Austria  High  Positive | RCT  92 physicians from the province of Salzburg  Group 1 (n=355): disease management program year one  Group 2 (n=335): RCT control group that later took part in disease management program year two  Group 3 (n=111): control group  Improve diabetes management | Context:  Quality improvement  Intervention:  Professional (education via workshop), Patient (education via workshop)  Theory:  No specified | Content: workshop for physicians and for their patients, templates for recording results of clinical/diagnostic tests in patient chart  Mode: in person, print material  Duration: 10 hours physicians, 9 hours patients  Frequency: Not specified  Audience: Physicians (training course) and their patients (patient education)  Personnel: Physician training by Austrian Diabetes Association, the Austrian Medical College, and the Austrian Society for General Practice; patient training by Working Group for Preventive Medicine Salzburg | Groups 1 and 2 showed significant improvement in HbA1c (p<0.001), cholesterol (p<0.001), triglycerides (p=0.001 for group 1 only), high density lipoprotein (p<0.001), and low density lipoprotein (p<0.001) |
| Reutens  2012 [40]  Australia  Low  No change | RCT  99 Asia-Pacific primary care physicians who each recruited 4 patients  INT (199 patients from 50 physicians): workshops, reminders, print materials, patient passports  CON (187 patients from 49 physicians): usual care  Improve diabetes management | Context:  Quality improvement  Intervention:  Professional (education via workshops, print material, reminders), Patients (print material)  Theory:  Not specified | Content: Workshops with presentations on guidelines and interactive discussion about implementation, reminder letters and desktop cards, checklist for patient charts, diabetes passports for patients to record results and prompt discussion with physicians  Mode: in person, print material  Duration: 12 months  Frequency: 2 workshops 3 months apart, 3 reminder letters  Audience: general practitioners  Personnel: not specified | HbA1c was not improved at 6 months (p = 0.340) and groups did not differ in control of other glycaemic indices, blood pressure or lipids after 6 or 12 months |
| Wallgren 2012 [41]  USA  Moderate  Positive | Observational  188 patients at the Womack Army Medical Center (98 pharmacist intervention; 90 standard care)  Improve diabetes management | Context:  New guideline  Intervention:  Patient (education via counselling, workshops)  Theory:  Not specified | Content: pharmacist visit to assess and discuss nutrition, workshops on nutrition, follow-up visit with pharmacist to assess progress  Mode: in person  Duration: 15 to 30 minute visit, 2-hour group classes  Frequency: 2 pharmacist visits, 2 workshops Audience: Diabetic patients  Personnel: Clinical pharmacists | Pharmacist group saw positive improvements in HbA1C (p<0.001); systolic (p=0.001) and diastolic (p=0.038) blood pressure (p=0.001 and 0.038); and low density lipoprotein (p=0.048) |
| Barcelo  2010 [42]  USA  Low  Positive | RCT  10 public health centres in the cities of Xalapa and Veracruz  INT (196 patients at 5 sites): Education sessions using the Breakthrough Series  CON (111 patients at five sites): usual care  Improve diabetes care delivery | Context:  Quality improvement  Intervention:  Professional (education via workshop), Patient (education via workshop)  Theory:  Not specified | Content: Professionals received training in patient diabetes education and foot care, and inservice training on diabetes management; referral system was modified to enable specialist to participate in patient consultations; an advisor visited intervention sites to provide case management support  Mode: in person  Duration: 18 months  Frequency: 3 training sessions  Audience: Primary care teams (physicians, nurses and other staff)  Personnel: not specified | Care was improved for INT patients for:   - glycemic control (28% to 39%, p<0.01) - cholesterol <65% to 76.5%, p<0.01) - foot examination (49% to 95%, p<0.01) - eye examination (10% to 73%, p<0.01) |
| Ciccone  2010 [43]  Italy  Moderate  Positive | Observational  20 primary care practices in Apulia Region (83 general practitioners and 1,160 patients)  Create collaborative teams and improve diabetes management | Context:  Quality improvement  Intervention:  Organizational (new role, multidisciplinary team), Patient (education via counselling, print material)  Theory:  Chronic Care Model | Content: care managers placed in primary care offices to promote multidisciplinary care and support patients to implement physician recommendations, patients received initial and follow-up assessments, individualized care plans, educational material on their specific condition, assistance with service coordination and one-on-one health counselling  Mode: in person, print material  Duration: 18 months  Frequency: not specified  Audience: general practitioners and their patients  Personnel: care managers (trained nurses) | - Increased self- efficacy, coping, ability access to social support and self-monitoring behaviour by 20% to 27% - Increased healthy diets from 39.4% to 80.7% of patients - Increased physical activity from 2.55 days to 4.18 days (p<0.0001) - Increased time for physical activity from 19.87 minutes to 32.90 (p<0.0001) - Decrease in body mass index, high density lipoprotein, and blood pressure and total cholesterol by at least 10% to 20% (p<0.0001) - Physical and mental health status was nearly 8.0 points above the national normal value of 47.6, and 5.3 points above pre-intervention score |
| Ena  2009 [44]  Spain  Low  Positive | Observational  138 patients at the internal medicine department in Hospital Marina Baixa  Improve diabetes management | Context:  Quality improvement  Intervention:  Professional (education via workshops, print material)  Theory:  Not specified | Content: workshop on glycaemia management plus interactive discussion on implementation, pocket guides, posters  Mode: in person, print material  Duration: 20-minute seminars over 1 month  Frequency: 10 seminars  Audience: Physicians and nurses in emergency and internal medicine departments  Personnel: Not specified | Significant reduction of administration of insulin from 50% to 3% (p=0.000) and median pre-discharge glycemic values (185mg/dL to 161 mg/dL (p=0.005) |
| Guzek  2009 [45]  USA  Moderate  Positive | Observational  1,592 patients from 12 primary care physicians from a group practice in Pennsylvania  Implement guideline and improve care delivery | Context:  New guideline  Intervention:  Professional (education via workshops, print material, reminders), Organizational (information technology), and Patient (print material)  Theory:  Not specified | Content: workshop featuring demonstrations of diabetes visit and motivational interviewing, self-study course and educational material, print material for patient education and referral forms to diabetes educators, electronic medical record checklist and reminder  Mode: in person, print material, computer application  Duration: not specified, study took place over 7 months  Frequency: single workshop  Audience: general practitioners, their patients  Personnel: self-study course developed by American College of Physicians, computer application developed by ACP Quality Committee with Physicians Health Alliance (PHA) | Significant improvement in HbA1c (p<0.001), low density lipoprotein cholesterol (p=0.033), urinary microalbumin (p=0.006), dilated eye exam (p=0.04), foot exam (p<0.001), blood pressure (p=0.003) and diabetes summary index (p<0.001) |
| Hahn  2008 [46]  USA  Low  Positive | Observational  1,016 patients from 54 family practices in New Jersey and Pennsylvania  Improve diabetes care delivery | Context:  Quality improvement  Intervention:  Professional (print material)  Theory:  Not specified | Content: checklist/reminder for patient charts  Mode: paper or electronic  Duration: 2 years  Frequency: not specified  Audience: Nurses  Personnel: study investigators | Flow sheets were used in 23% of medical records and use was associated with mean guideline adherence score for assessment of diabetes (55.38 sheets used vs 50.13 sheets not used, p=0.02), and treatment of diabetes (79.59 sheets used vs 74.71 sheets not used, p=0.004) |
| Rothe  2008 [47]  Germany  Moderate  Positive | Observational  291,771 patients from 2,028 general practitioners and 102 diabetes specialists in Saxony, Germany  Improve physician collaboration and diabetes management | Context:  New guideline  Intervention:  Professional (guidelines, education via workshops, reimbursement), Organizational (integration of services), Patient (education via counselling)  Theory:  Not specified | Content: workshops on guidelines and interim study findings, integrated practice guidelines specifying criteria for referral to diabetes specialized practitioners (DSPs), integrated diabetes management structure to establish relationship between general practitioners and DSPs, payment of 6 Euros/patients to general practitioners and DSPs for care coordination; patient education program including lifestyle counselling Mode: in person, guidelines, structure, payment for professionals; in person for patients  Duration: not specified, study over 3 years  Frequency: 2-4 workshops per year for professionals; patients not specified  Audience: general practitioners and specialists  Personnel: guideline developed by Saxon Diabetes Committee, professional and patient counselling by DSPs | - Median and mean HbA1C decreased from 8.5 to 7.5% - 78% achieved therapeutic targets according to guidelines compare with baseline (69%) - 61% achieved blood pressure <140/90 mmHg vs 50% at baseline - Patients with poorly controlled diabetes benefited the most (p<0.001) |
| Davies  2008 [48]  Canada  High  Positive | Observational  1,273 patients and 95 nurses in eleven health care organizations  Improve diabetes care delivery (study also examined other conditions; diabetes relevant data reported here) | Context:  New guidelines  Intervention:  Professional (print material, education, identify barriers, incentive not tied to performance)  Theory:  Not specified | Content: workshops to present guideline recommendations, paid training session on various guideline implementation strategies and use of an implementation toolkit, follow-up teleconferences to discuss implementation problems and solutions  Mode: in person, telephone, print material  Duration: two-hour implementation training  Frequency: single workshop, training session and teleconference  Audience: nurses  Personnel: coordinated by Registered Nurses Association of Ontario with presentations by leaders of guideline development panels and clinical resource nurses | - Statistically significant improvements found in at least 50% of indicators for asthma (52%), diabetes foot care (83%), and venous leg ulcers (60%) - Little change in indicators for breast feeding, delirium-dementia-depression and smoking cessation - Organizations with improvements reported two unique implementation strategies - hands-on skill practice sessions for nurses and development of new patient education materials |
| Sipila  2008 [49]  Finland  Moderate  Positive | Observational  62 program facilitators (general practitioners and nurses) in multiple Finnish health stations  Improve clinician collaboration, and diabetes care delivery and management | Context:  Quality improvement  Intervention:  Professional (tailor guidelines, education, self-audit, feedback, local facilitators, reimbursement, incentive tied to compliance), Organizational ( multidisciplinary teams)  Theory:  Not specified | Content: two voluntary facilitators (paid a small monthly stipend and motivated by competition for most improved site) at each site took part in educational sessions during which they tailored guidelines to suit local multi-disciplinary processes, and learned about audits, implementation, how to give feedback, use of quality tools, leadership and change management; all staff performed self-audit with support from facilitators  Mode: in person, print and electronic guidelines  Duration: 2 years  Frequency: 16 educational sessions for facilitators, self-audit performed by all staff at one year  Audience: Staff at each site  Personnel: one doctor and nurse from each site (facilitator pair) | Management improved for blood pressure (from 17% to 22%), diabetes control (from 31% to 34%) and dyslipidemia (71% to 64%) |
| Jones  2006 [50]  USA  Moderate  Positive | Observational  6 physicians in a single family medicine clinic (58 patients diabetes management system, 115 patients usual care)  Improve diabetes care delivery and management | Context:  Quality improvement  Intervention: Professional (reminders, feedback), Organizational (information technology)  Theory:  Not specified | Content: personal digital assistant (PDA) in which guideline recommendations are embedded tracks patient details, visits and results, offers reminders and generates quarterly reports that are sent to patient and added to medical record  Mode: internet  Duration: 16 months  Frequency: not specified  Audience: family physicians  Personnel: Not specified | Two of four processes improved among intervention patients: foot exam: 50 vs 8 (p<0.0001) and eye exam: 46 vs 12 (p<0.0001). They did not experience improved outcomes (HbA1c, blood pressure, fasting plasma glucose, low density lipoprotein, high density lipoprotein, triglycerides) |
| Siminerio  2005 [51]  USA  Moderate  Positive | Observational  104 patients and 6 physicians in a rural primary care practice  Improve diabetes management | Context:  Quality improvement  Intervention:  Professional (education, print material), Patient (education, reminder)  Theory:  Chronic Care Model | PHYSICIANS  Content: workshop on guidelines, routine clinical exams, diabetes self-management education for patients; given barriers to diabetes care instrument  Mode: in person, print material  Duration: 12 months  Frequency: single workshop  Audience: physicians  Personnel: diabetes educators  PATIENTS  Content: group self-management sessions on goal-setting, behavioural change, and nutrition; follow-up phone call to discuss progress  Mode: in person, telephone  Duration: 2 hour group sessions over 12 months  Frequency: 5 sessions (biweekly)  Audience: 17 of the 104 patients in participating physician practices  Personnel: diabetes educators; dietitian | - Adherence to all measured processes improved significantly (HbA1c, lipid profile, urinalysis, dilated eye exam, foot exam, monofilament) - HbA1c (p=0.007), low density lipoprotein (p=0.01) and high density lipoprotein (p=0.05) significantly improved while blood pressure did not |
| Dijkstra  2005 [52]  Netherlands  Low  Positive | RCT  13 Dutch general hospitals  INT1 (n=4): professional program  INT2 (n=4): patient program  CON (n=5): usual care  Assess cost-effectiveness of two programs | Context:  Quality improvement  Intervention:  Professional (education, reminders, feedback), Patient (education, print material)  Theory:  Not specified | Content: INT1 - workshops for physicians and nurses, desktop reminder cards, feedback at six months; INT2 – similar to INT1plus instruction on use of a patient diabetes passport, workshop for patients, brochures/posters for waiting room  Mode: in person, print material, feedback not specified  Duration: not specified, study over 1 year  Frequency: single workshop  Audience: physicians, nurses, patients  Personnel: opinion leader (not specified) | - HbA1c decreased by 0.1% in INT1 and 0.3% in INT2, and increased by 0.2% in control group (p<0.001) - Life expectancy improved by 0.34 (INT1) and 0.63 (INT2) years, and quality-adjusted life years by 0.29 (INT1) and 0.59 (INT2) - Incremental cost was 32,218 Euro for professional program and 16,353 for patient program compared with control, and 881 Euro for patient program compared with professional program |
| O’Connor  2005 [53]  USA  Low  Positive | Observational  122 patients in one electronic medical record (n=57) and one non-electronic medical record (n=65) clinic in a Minnesota medical group  Improve diabetes management | Context:  Quality improvement  Intervention: Professional (reminders), Organizational (information technology)    Theory:  Not specified | Content: computer decision support system linked to medical record showed test results, prompts and reminders, and ordered pharmaceuticals; ongoing one-on-one support provided to physicians on use of system  Mode: computer/electronic, support not specified  Duration: 5 years  Frequency: ongoing  Audience: multi-specialty physicians  Personnel: Not specified | - Number of HbA1c tests performed in the EMR clinic compared with control clinic increased significantly at 2 years (p<0.04) and 4 years (p<0.001) - HbA1c levels improved in both clinics (p <0.05) with no significant differences between clinics at 2 years (p=0.10) or 4 years (p=0.27) |
| Abbasi  2004 [54]  USA  Low  Positive | Observational  613 patients in specialized diabetes centres and primary care practices in Michigan (119 control subjects)  Improve diabetes management | Context:  Quality improvement  Intervention:  Patient incentives based on compliance  Theory:  Not specified | Content: patients undergo pre-consultation test on diabetes knowledge and nutrition, those failing test referred to diabetes education program, enrolled in counselling sessions with a diabetes educator, or provided literature to study and retake test, credits awarded for test results and achieving lifestyle behaviours and target clinical test results  Mode: in person, print material  Duration: not specified  Frequency: test offered annually at clinic visits  Audience: patients with diabetes  Personnel: provider scores test and initiates credit system | Compared with controls, intervention patients achieved improved HbA1c (p<0.001), blood pressure (p<0.007) and serum lipids (p<0.01) |
| Zgibor  2004 [55]  USA  Low  Positive | Observational  208 patients from 5 primary care practices and one specialty diabetes clinic in Pittsburgh  Improve diabetes care delivery and management | Context:  Quality improvement  Intervention:  Professional (educational sessions, guideline material) and Patient (educational sessions)  Theory:  Not specified | Content: workshop on diabetes management and complications; recommendations of test frequency and target measures; diabetes nurse educators visited practices to conduct self-management education sessions for patients, provide wallet card of diabetes care goals  Mode: in person, printed materials  Duration: one day workshop, 1 year  Frequency: single workshop; biweekly visits  Audience: Physicians and office staff  Personnel: endocrinologist, nurses, nurse diabetes educators | 100% had annual foot examinations, 91.8% had annual creatinine, and 85.1% had 2 annual HbA1c tests |

**Heart Disease** Studies

| Study  Country  Risk of bias  Overall impact | Research Design  Participants  Objective | Implementation Approach | Implementation Design | Impact |
| --- | --- | --- | --- | --- |
| Willens  2013 [56]  USA  Moderate  No change | Observational  529 patients seen by cardiologists at the University of Miami Health System  Improve knowledge about and use of stress echocardiography (SE) | Context:  Quality improvement  Intervention:  Professional (presentation, print material, reminder)  Theory:  Not specified | Content: lecture on use of SE, printed material on indications for SE, email reminder  Mode: in-person, print material, email reminder  Duration: one-hour lecture, email reminder at one week  Frequency: single lecture and reminder  Audience: 15 (54%) of 28 member cardiologists attended the lecture  Personnel: Not specified | No significant change in appropriateness ratings of SEs requested before and after the intervention (p=0.497) |
| Aziz  2011 [57]  USA  High  Positive | Observational  Clinicians and staff at St.Luke’s-Roosevelt Hospital, New York City  Improve management of cardiac patients | Context:  Quality improvement  Intervention:  Professional (lectures, print material, educational workshops, feedback)  Theory:  Not specified | Content: lectures, posters featuring 8 critical pathways posted in key hospital areas, pocket cards featuring pathways, implementation workshops, information on web site, feedback reports on use of pathways  Mode: in person, print, web site  Duration: four years  Frequency: not specified  Audience: Physicians, nurses, staff  Personnel: clinical task force developed pathways and offered lectures | - Pathway-prescribed drug utilization at discharge increased from baseline: ASA from 83% to 91%, Beta blocker from 61% to 87%, ACE inhibitors or angiotension receptor blockers from 51% to 71%, statins from 63% to 75% (p<0.0001) over four-year study period - Decreased re-admissions (36.5% to 14.5%, p < 0.001) - Decreased presence of angina-free symptoms (12% to 3.1%, p < 0.001) - Lower incidence of co-morbidities and mortality (8% to 1.8%, p < 0.001) |
| Goud  2009 [58]  Netherlands  Low  Positive | RCT  2,787 patients from 21 Dutch cardiac rehabilitation centres  INT (16 sites) decision support system with embedded guideline recommendations  CON (15 sites): decision support system  Improve use of cardiac rehabilitation | Context:  New guideline  Intervention:  Structural (information system, reimbursement, training)  Theory:  Not specified | Content: reimbursed for purchasing system, training session, free helpdesk services  Mode: In-person training session  Duration: 6 months  Frequency: 1 session  Audience: Multidisciplinary healthcare teams  Personnel: Not specified | Recommended rehabilitation improved (adjusted difference, 95% CI):   - exercise therapy by 3.5 (0.1 to 5.2) - education therapy by 23.7 (15.5 to 29.4) - relaxation therapy by 41.6 (25.2 to 51.3) |
| Lainscak  2004 [59]  Slovenia  Moderate  Positive | Observational  50 patients in the Murska Sobota General Hospital heart failure clinic  Improve patient knowledge, pharmacological management and quality of life | Context:  Quality improvement  Intervention:  Patient (education, counselling, print material)  Theory:  Not specified | Content: 45 minute physician consultation about heart failure management and compliance, print summary (first visit), 10 minute nurse counselling on self-management (subsequent visits)  Mode: in-person, print  Duration: 1 consultation, 2 counselling sessions  Frequency: 3 visits over 1 year  Audience: Patients with symptoms and signs of heart failure, left ventricular ejection fraction <45%, and visited clinic at least 3 times  Personnel: Physicians (initial consultation) and nurses (counselling) | - Patient knowledge about their condition improved after two visits to heart failure clinic from 4.8 (1.5) to 7.3 (1.4), p<0.001 - Pharmacological management improved for Beta blocking agents (from 40% to 84% of patients, p<0.001), monotherapy (reduced from 32% to 2% of patients, p<0.001), and use of three neurohormonal drugs (from 22% to 64% of patients, p<0.001) - Quality of life scores improved from 3.7 (1.0) to 5.3 (1.1), p <0.001) - Health scores improved from 3.7 (1.1) to 5.1 (1.1), p<0.001 |
