# Supplementary material for: Trends in guideline implementation: a scoping systematic review
Source: Implement Sci. 2015 Apr 21;10:54. doi: 10.1186/s13012-015-0247-8 (PMC4409784; doi:10.1186/s13012-015-0247-8)
Supplement: Additional file 4: — Methodological assessment of eligible studies. Scoring of randomized control trials and observational studies using the Cochrane Risk of Bias tool and Down’s and Black Checklist, respectively [38-40,42,58,52,34,28,35-37,56,32,41,57,29,43-45,30,48,46,47,49,50,33,53,31,51,54,59,55 ]. [file 13012_2015_247_MOESM4_ESM.doc]

**Additional File 4. Methodological assessment of eligible studies**

Randomized controlled trials (Cochrane Risk of Bias tool)

| Study | Sequence  generation | Allocation  concealment | Blinding of participants or personnel | Incomplete outcome data | Selective outcome reporting | Other sources of bias | Summary assessment |
| --- | --- | --- | --- | --- | --- | --- | --- |
| Steyn 2013 [38] | low | low | low | low | low | low | low risk of bias |
| Flamm 2012 [39] | high | high | low | high | low | high | high risk of bias |
| Reutens 2012 [40] | low | uncertain | low | low | low | uncertain | low risk of bias |
| Barcelo 2010 [42] | low | low | low | low | low | low | low risk of bias |
| Goud 2009 [58] | low | low | low | high | low | low | low risk of bias |
| Dijkstra 2005 [52] | low | uncertain | low | low | low | uncertain | low risk of bias |
| Myers 2004 [34] | low | uncertain | low | low | low | low | low risk of bias |

Observational studies (Down’s and Black Checklist)

| **Study** | **1** | **2** | **3** | **4** | **5** | **6** | **10** | **11** | **12** | **13** | **18** | **20** | **21** | **22** | **25** | **Risk of Bias** |
| --- | --- | --- | --- | --- | --- | --- | --- | --- | --- | --- | --- | --- | --- | --- | --- | --- |
| Brosseau 2013 [28] | 1 | 1 | 1 | 1 | 0 | 1 | 1 | 1 | 1 | 1 | 1 | 1 | 1 | 1 | 0 | Moderate |
| Butala 2013 [35] | 1 | 1 | 1 | 1 | 0 | 1 | 1 | 1 | 1 | 0 | 1 | 1 | 1 | 1 | 0 | Moderate |
| Hager 2013 [36] | 1 | 1 | 1 | 1 | 0 | 1 | 1 | 1 | 1 | 1 | 1 | 1 | 1 | 1 | 0 | Moderate |
| Kuhne-Eversmann 2013 [37] | 1 | 1 | 1 | 1 | 0 | 1 | 1 | 1 | 1 | n/a | 1 | 1 | 1 | 1 | 1 | High |
| Willens 2013 [56] | 1 | 1 | 1 | 1 | 0 | 1 | 1 | 1 | 1 | 1 | 1 | 1 | 1 | 0 | 0 | Moderate |
| Jani 2012 [32] | 1 | 1 | 1 | 1 | 0 | 1 | 1 | 1 | 1 | 1 | 1 | 1 | 1 | 1 | 0 | Moderate |
| Wallgren 2012 [41] | 1 | 1 | 1 | 1 | 0 | 1 | 1 | 1 | 1 | 1 | 1 | 1 | 1 | 1 | 0 | Moderate |
| Aziz 2011 [57] | 1 | 0 | 0 | 1 | 0 | 1 | 1 | n/a | n/a | 1 | 1 | 1 | n/a | n/a | 0 | High |
| Lineker 2011 [29] | 1 | 1 | 1 | 1 | 0 | 1 | 1 | 1 | 1 | 1 | 1 | 1 | 1 | 1 | 0 | Moderate |
| Ciccone 2010 [43] | 1 | 1 | 1 | 1 | 0 | 1 | 1 | 1 | 1 | 1 | 1 | 1 | 1 | 1 | 0 | Moderate |
| Ena 2009 [44] | 1 | 1 | 1 | 1 | 0 | 1 | 1 | 1 | 1 | 1 | 1 | 1 | 1 | 1 | 1 | Low |
| Guzek 2009 [45] | 1 | 1 | 1 | 1 | 0 | 1 | 1 | 1 | 1 | 0 | 1 | 1 | 1 | 1 | 0 | Moderate |
| Laine 2009 [30] | 1 | 1 | 1 | 1 | 0 | 1 | 1 | 1 | 1 | 1 | 1 | 1 | 1 | 1 | 0 | Moderate |
| Davies 2008 [48] | 1 | 1 | 1 | 1 | 0 | 1 | 1 | 1 | 1 | 1 | 0 | 1 | 0 | 0 | 1 | High |
| Hahn 2008 [46] | 1 | 1 | 1 | 1 | 1 | 1 | 1 | 1 | 1 | 1 | 1 | 1 | 1 | 1 | 1 | Low |
| Rothe 2008 [47] | 1 | 1 | 1 | 1 | 0 | 1 | 1 | 1 | 1 | 1 | 1 | 1 | 1 | 1 | 0 | Moderate |
| Sipila 2008 [49] | 1 | 1 | 1 | 1 | 0 | 1 | 0 | 1 | 1 | 1 | 1 | 1 | 1 | 1 | 0 | Moderate |
| Jones 2006 [50] | 1 | 1 | 1 | 1 | 0 | 1 | 1 | 1 | 1 | 1 | 1 | 1 | 1 | 1 | 0 | Moderate |
| White 2006 [33] | 1 | 1 | 1 | 1 | 0 | 1 | 1 | 1 | 1 | 1 | 1 | 1 | 1 | 1 | 0 | Moderate |
| O'Connor 2005 [53] | 1 | 1 | 1 | 1 | 1 | 1 | 1 | 1 | 1 | 1 | 1 | 1 | 1 | 1 | 1 | Low |
| Rahme 2005 [31] | 1 | 1 | 1 | 1 | 1 | 1 | 1 | 1 | 1 | 1 | 1 | 1 | 1 | 1 | 1 | Low |
| Siminerio 2005 [51] | 1 | 1 | 1 | 1 | 0 | 1 | 1 | 1 | 1 | 1 | 1 | 1 | 1 | 1 | 0 | Moderate |
| Abbasi 2004 [54] | 1 | 1 | 1 | 1 | 0 | 1 | 1 | 1 | 1 | 1 | 1 | 1 | 1 | 1 | 1 | Low |
| Lainscak 2004 [59] | 1 | 1 | 1 | 1 | 0 | 1 | 1 | 1 | 1 | 1 | 1 | 1 | 1 | 1 | 0 | Moderate |
| Zgibor 2004 [55] | 1 | 1 | 1 | 1 | 1 | 1 | 1 | 1 | 1 | 1 | 1 | 1 | 1 | 1 | 1 | Low |

1. Is the hypothesis/aim/objective of the study clearly described?
2. Are the main outcomes to be measured clearly described in the Introduction or Methods section?
3. Are the characteristics of the patients included in the study clearly described?
4. Are the interventions of interest clearly described?
5. Are the distributions of principal confounders in each group of subjects to be compared clearly described?
6. Are the main findings of the study clearly described?

10. Have actual probability values been reported (e.g. 0.035 rather than <0.05) for the main outcomes except where the probability value is less than 0.001?

11. Were the subjects asked to participate in the study representative of the entire population from which they were recruited?

12. Were those subjects who were prepared to participate representative of the entire population from which they were recruited?

13. Were the staff, places, and facilities where the patients were treated, representative of the treatment the majority of patients receive?

18. Were the statistical tests used to assess the main outcomes appropriate?

20. Were the main outcome measures used accurate (valid and reliable)?

21. Were the patients in different intervention groups (trials and cohort studies) or were the cases and controls (case-control studies) recruited from the same population?

22. Were study subjects in different intervention groups (trials and cohort studies) or were the cases and controls (case-control studies) recruited over the same time?

25. Was there adequate adjustment for confounding in the analyses from which the main findings were drawn?
